# The Expanded Central Dogma: Genome Resynthesis, Orthogonal Biosystems, Synthetic Genetics

**DOI:** 10.1146/annurev-biophys-111622-091203

**Published:** 2023-05-09

**Authors:** Karola Gerecht, Niklas Freund, Wei Liu, Yang Liu, Maximilian J.L.J. Fürst, Philipp Holliger

**Affiliations:** 1 MRC Laboratory of Molecular Biology, Cambridge Biomedical Campus, Cambridge, United Kingdom; 2 Current address: Groningen Biomolecular Sciences and Biotechnology Institute, University of Groningen, Groningen, Netherlands

**Keywords:** synthetic biology, orthogonality, genome synthesis, synthetic genetics, XNA, unnatural base pairs

## Abstract

Synthetic biology seeks to probe fundamental aspects of biological form and function by construction [i.e., (re)synthesis] rather than deconstruction (analysis). In this sense, biological sciences now follow the lead given by the chemical sciences. Synthesis can complement analytic studies but also allows novel approaches to answering fundamental biological questions and opens up vast opportunities for the exploitation of biological processes to provide solutions for global problems. In this review, we explore aspects of this synthesis paradigm as applied to the chemistry and function of nucleic acids in biological systems and beyond, specifically, in genome resynthesis, synthetic genetics (i.e., the expansion of the genetic alphabet, of the genetic code, and of the chemical make-up of genetic systems), and the elaboration of orthogonal biosystems and components.

## 1 Introduction

The intricate molecular machinery of living cells and organisms is controlled by a central information system based on the nucleic acids DNA and RNA, as conceptualized in the central dogma of molecular biology. In this review, we seek to provide an overview of current synthetic biology approaches to engineering, controlling, and expanding the central dogma via use of synthetic approaches for the reassignment of the informational units and exploration of alternative chemistries for their ability to support life or provide expanded functionalities.

**Figure 1:**
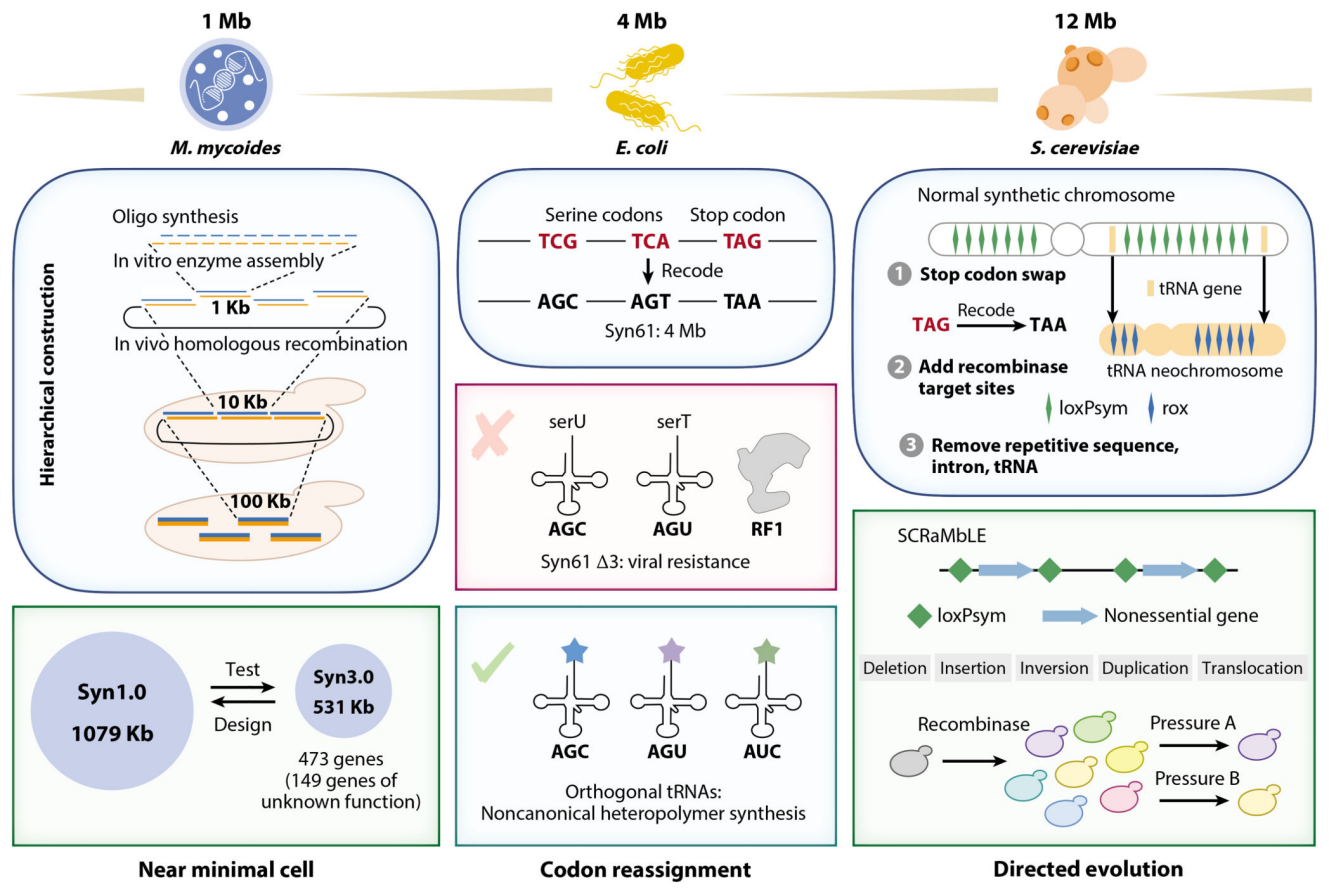
Synthetic genomes: design, construction, and repurposing. Three main genome resynthesis projects have been applied in *Mycoplasma mycoides, Escherichia coli*, and *Saccharomyces cerevisiae*, respectively, with sizes ranging from 1 Mbp to 12 Mbp. Work on Syn1.0 established the hierarchical construction workflow to synthesize the *M. mycoides* genome, from oligonucleotides to larger fragments that were eventually assembled into the 1 Mbp genome. With the bottom-up method, a genome of half the size—Syn3.0—was constructed to study what genes are essential for maintaining a near-minimal cell. In the synthetic genome for *E. coli*, two serine codons and a stop codon were removed and replaced by their isoforms, resulting in the Syn61 strain with 61 codons. Further deletion of the corresponding tRNAs enabled resistance to viral infection and provided orthogonal tRNAs with space for heteropolymer synthesis. The synthetic yeast project includes the swap of a stop codon, removal of repetitive elements, and addition of recombinase recognition sites. Various rearrangements and genome diversity can be generated by SCRaMbLE, enabling directed evolution of the synthetic strain for downstream applications. Abbreviations: RF1, release factor 1; tRNA, transfer RNA.

Arguably the biggest impact of such synthetic approaches has come from the advances enabled by the ever-increasing efficiency and scale of the classic phosphoramidite DNA synthesis of Caruthers (13). Together with more sophisticated DNA assembly methods, these advances have begun to enable the engineering and wholesale rewriting of genetic information at the genome level (Figure 1). We discuss recent advances in genome resynthesis specifically for codon reassignment. Furthermore, we highlight possible routes toward the generation of biosystems whose genomes and/or their readout are informationally or chemically orthogonal, providing a genetic firewall against unwanted release of engineered organisms.

## 2 Genome Synthesis

In 2010, the J.C. Venter Institute successfully transplanted an entirely chemically synthesized genome of *Mycoplasma mycoides* to a *Mycoplasma capricolum* recipient cell, which then developed into *M. mycoides* progeny; this experiment conclusively established the primacy of genotype in determining phenotype (35). This pioneering work established the feasibility of artificial genome synthesis and introduced several technological advances, such as the hierarchical in vitro and in vivo assembly strategy from which subsequent synthetic genome projects benefited, including the minimal genome JCVI-syn3.0 (531 kbp, truncated from the JCVI-syn1.0 of 1,079 kbp) (47). Importantly, when—during the design-build-test cycle—it was found that not all of the (proposed) nonessential genes could be deleted, the synthesis and assembly platform technology enabled a systematic study and evaluation of the essential genes and encoded functions that appeared to be essential to maintaining cellular life.

Two other powerful applications of whole-genome synthesis (or rewriting) are genetic code expansion (GCE) and reassignment, both of which have the ultimate aim of converting cellular protein translation into a general system for the sequence-defined synthesis of artificial polymers. GCE originally relied on amber suppression, based on the pioneering work of Schultz and colleagues (117). This strategy requires aminoacyl-transfer RNA (tRNA)-synthetase (aaRSx)–tRNA (tRNAx) pairs that are (*a*) orthogonal (x) to the tRNAs and aaRSs in the target organism (i.e., are not charged by a noncognate aaRS or do not charge noncognate tRNAs), (*b*) engineered for the orthogonal aaRSx to load a defined unnatural amino acid (uAA) (X) onto the tRNAx, and (*c*) engineered so that the tRNAx recognizes a stop codon, thus in effect reassigning the stop codon to the uAA. GCE has undergone many improvements and expansions with regard to the types of amino acids that are suitable for incorporation, resulting in widespread applications (16). However, the scope of amber suppression is inherently limited by competition with the natural (stop) function. Engineering of release factor 1 (RF1) (96) or the ribosome for enhanced amber suppression (116) can alleviate some of these issues.

Genome-wide codon reassignment, which relies on the genetic engineering or de novo synthesis of organisms with a compressed genetic code, has been transformative for the field. By systematically replacing target codons with synonymous codons, the degeneracy of the genetic code can be leveraged to generate synthetic organisms with free codons. Initially, multiplex automated genome engineering (MAGE) was used to remove all amber codons from the *Escherichia coli* genome by iterative mutagenesis and the deletion of release factor 1 (RF1) (48) to free up the amber codon for reassignment. More recently, replicon excision for enhanced genome engineering through programmed recombination (REXER) (115), an advanced new method for genome engineering, enabled the rational identification of specific code compression schemes and the resynthesis of the 4-Mbp genome of *E. coli* (31) (Figure 1), freeing up three codons for reassignment (89).

Wholesale genome resynthesis is also advancing at pace in a eukaryotic organism. The Sc 2.0 project aims to resynthesize the chromosomes of the yeast *Saccharomyces cerevisiae*, applying three design principles: TAG stop codons are swapped to TAA, and synonymous mutations are introduced to generate distinguishing watermarks; repetitive elements and introns are removed, while the tRNA genes are relocated to create the plus one neochromosome; and recombinase-recognizable loxPsym sites are added between nonessential genes to enable genome rearrangement and evolution (the SCRaMbLE system) (25). By 2017, 6.5 chromosomes had been synthesized separately, and approximate wild-type phenotypes were observed with these strains (88). The SCRaMbLE system showed its power as a directed evolution platform to evolve yeast strains with improved fitness and productivity (10, 62). The complete synthesis of the 16 yeast chromosomes in a whole synthetic Sc 2.0 strain could also be combined with other intriguing work that fused all chromosomes into one (100) to test the limits of genomic engineering in yeast. All of these advances have spurred the even more ambitious undertaking of a “human genome project-write” for human genome synthesis (11). The effective implementation of genome resynthesis and engineering at this scale will likely require significant advances in high-fidelity DNA synthesis and the handling of even larger DNA fragments for the construction of gigabase-scale genomes. On a smaller scale, the power of synthetic genomics was illustrated in the rapid (1-week) assembly of the complete genome of SARS-CoV-2 (111).

## 3 Synthesis Of Orthogonal Biosystems

Genome resynthesis provides an opportunity for the wholesale reconfiguration of biological systems, for example, in the pursuit of orthogonal biosystems, i.e., systems that are sufficiently decoupled from natural or host organism biology that they can function and evolve independently (59). In light of the ever-increasing possibilities and extent of genome engineering and modification, possible effects of an unplanned release of engineered lifeforms into the environment and their potential interactions with natural life require serious consideration (112). Thus, the establishment of effective safety measures is of utmost importance. Orthogonal biosystems, as defined above, which rely on divergent genetic information and a dedicated set of engineered components for propagation and/or expression, together with engineered differences in metabolism or the reliance on building blocks not found in nature, have great potential as biosafety systems, as orthogonality is not easily reverted by mutational drift.

Orthogonal genetic elements such as T7 RNA polymerase have been used for decades, often merely for the sake of convenience in the absence of cross-reactivity with the host chassis; the same motivation sparked the application of an orthogonal hypermutation-enabling DNA polymerase in yeast (85). A broader motivation for orthogonality, however, arises from the desire to prevent biological interaction on an ecological scale and thereby achieve pathogen resistance and/or biocontainment of non-natural biological systems (70, 95).

### 3.1 Semantic Orthogonality

**Figure 2:**
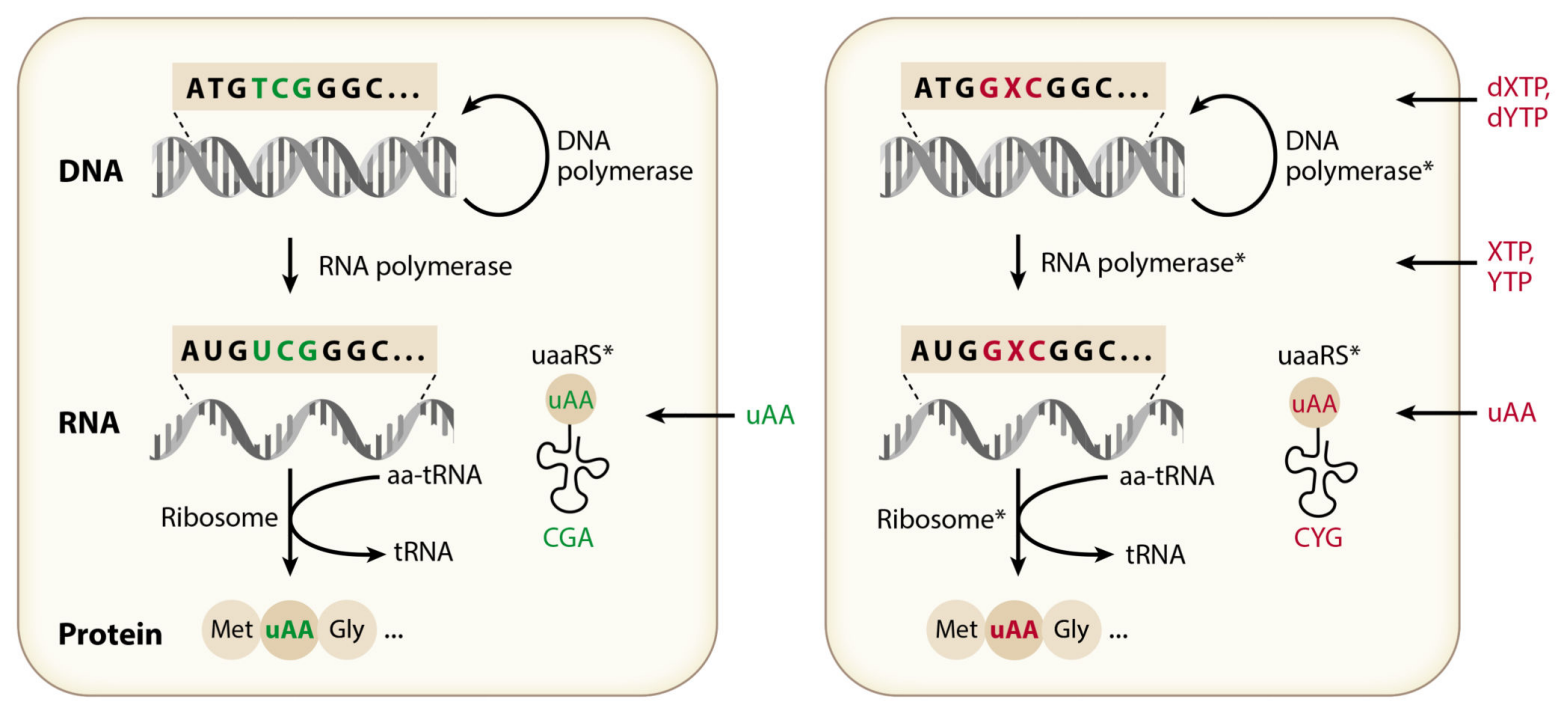
Semantic versus alphabetic orthogonality. In semantic orthogonality, after genome resynthesis with code compression, an orphan codon (TCG, previously coding for Ser) is reassigned to an unnatural amino acid (uAA) (*left*) (unnatural or engineered components are designated with *). Alphabetic orthogonality requires genetic alphabet expansion by genomic insertion, replication, and transcription of a UBP X:Y (creating the new codon dGXC) and a CYG anticodon transfer RNA (tRNA). This also requires import of (d)XTP [(d)YTP] for replication (transcription), as well as potentially (engineered, marked by *) DNA and RNA polymerases and ribosome (*right*). Both require an (engineered) orthogonal tRNA synthetase (uaaRS*):tRNA pair assigned to the new codon and uAA import.

Because virtually all strategies for the prevention of unintentional environmental proliferation (56) can theoretically be escaped by mutational drift and evolution, successful strategies must aim for the minimization of the likelihood of evolutionary escape. While biosafety systems such as kill switches or metabolic auxotrophy may be overcome by only a few mutations or gene transfer events, reconfiguration of the genetic system, e.g., by genetic code reassignment (semantic orthogonality) (Figure 2; see also Figure 4 below) potentially enables a virtually unbreachable informational firewall (86).

A key step toward the achievement of semantic orthogonality was the development of orthogonal ribosomes (o-ribs). These allow translation of dedicated orthogonal messenger RNAs (mRNAs) (not translated by the wild-type ribosome), which in turn frees up the o-rib for engineering without toxicity effects. This strategy has enabled the evolution of o-ribs with enhanced amber suppression (83, 116) or an ability to translate four base (quadruplet) codons, which can potentially free up new codon space for assignment of more than one uAA (77). In eukaryotes, orthogonal translation has even been shown to be implemented as a function of spatial partitioning within engineered membraneless organelles (87).

Resynthesis and codon reassignment of the *E. coli* genome can aid in both genetic code expansion and the establishment of orthogonality. Going beyond single (amber) codon removal and synthetic auxotrophy by establishing cell dependence on uAAs (69, 92), genome resynthesis with code compression has provided a striking validation of the potential of semantic orthogonality. By (*a*) replacing two isoacceptor serine codons, TCG and TCA, and the TAG stop codon with the synonymous codons AGC, AGT, and TAA, respectively, and (*b*) concurrently removing the corresponding serine tRNAs and RF1, three new codons were freed up for genetic code expansion. This gave rise to an organism (Syn61Δ3) with a compressed 61-codon genetic code (31) that is orthogonal to gene transfer (e.g., phage infection) from the 64-codon biosphere (89). However, it could be argued that its orthogonality remains one sided, as gene transfer from Syn61 to the biosphere is in principle still possible. Complete genetic isolation could for instance be achieved via reassignment of the vacant codons to three new uAAs, obtaining a new divergent genetic code.

Another form of such semantic orthogonality (Figure 2) may involve the (partial) rewriting of the code by codon expansion to quadruplet codons, potentially enabling a 256-codon code. While there appear to be no fundamental obstacles to the translation of at least some quadruplet codons within mixed triplet codes or in series (19, 22), the need for the elaboration of large numbers of mutually orthogonal quadruplet tRNA–aaRS pairs, as well as the issue of avoidance of code ambiguity (i.e., triplet or quadruplet register), poses obstacles to the expanded use of quadruplet codons.

In the future, a larger number of reassigned codons localized in essential genes, together with orthogonal translation that is dependent on the supply of uAAs or uAA precursors (93), should provide a near-unbreachable informational firewall between the natural and orthogonal genetic systems and thus a large degree of genetic isolation from the biosphere. Below, we discuss other opportunities for orthogonality based on expanded chemistries providing informational, chemical, or stereochemical divergence.

### 3.2 Alphabetic Orthogonality: Unnatural Base Pairs

While genome resynthesis with code compression may also be leveraged to reduce ambiguity in quadruplet translation, alternative approaches such as the synthesis and introduction of new orthogonal base pairs [also called unnatural base pairs (UBPs)] constitute a promising alternative (Figure 2). Indeed, genetic alphabet expansion by one or two orthogonal new base pairs potentially yields as many as 216 (1 UBP) or 512 (2 UBPs) new triplet codons (and thousands of potential quadruplet codons). Even considering likely codon degeneracy, such an approach would potentially enable an explosive expansion of the chemical repertoire of translation.

However, major obstacles must be overcome to realize this vision: (*a*) The unnatural nucleotide bases and their activated triphosphates need to be available in the cell at sufficiently high levels without causing toxicity (i.e., they must be orthogonal at both the nucleoside and nucleotide level); (*b*) DNA and RNA polymerase(s) must enable their replication and transcription with high efficiency and fidelity; and (*c*) the ribosome must be able to correctly translate the new UBP-containing codons to incorporate a defined amino acid into the growing polypeptide chain, which also requires the presence of UBP-tRNAs and cognate aaRSs capable of loading UBP-tRNA specifically with the appropriate uAA (Figure 2). The encountered challenges are paradigmatic of the general introduction of expanded chemistries into organisms and are therefore discussed in detail below.

**Figure 3:**
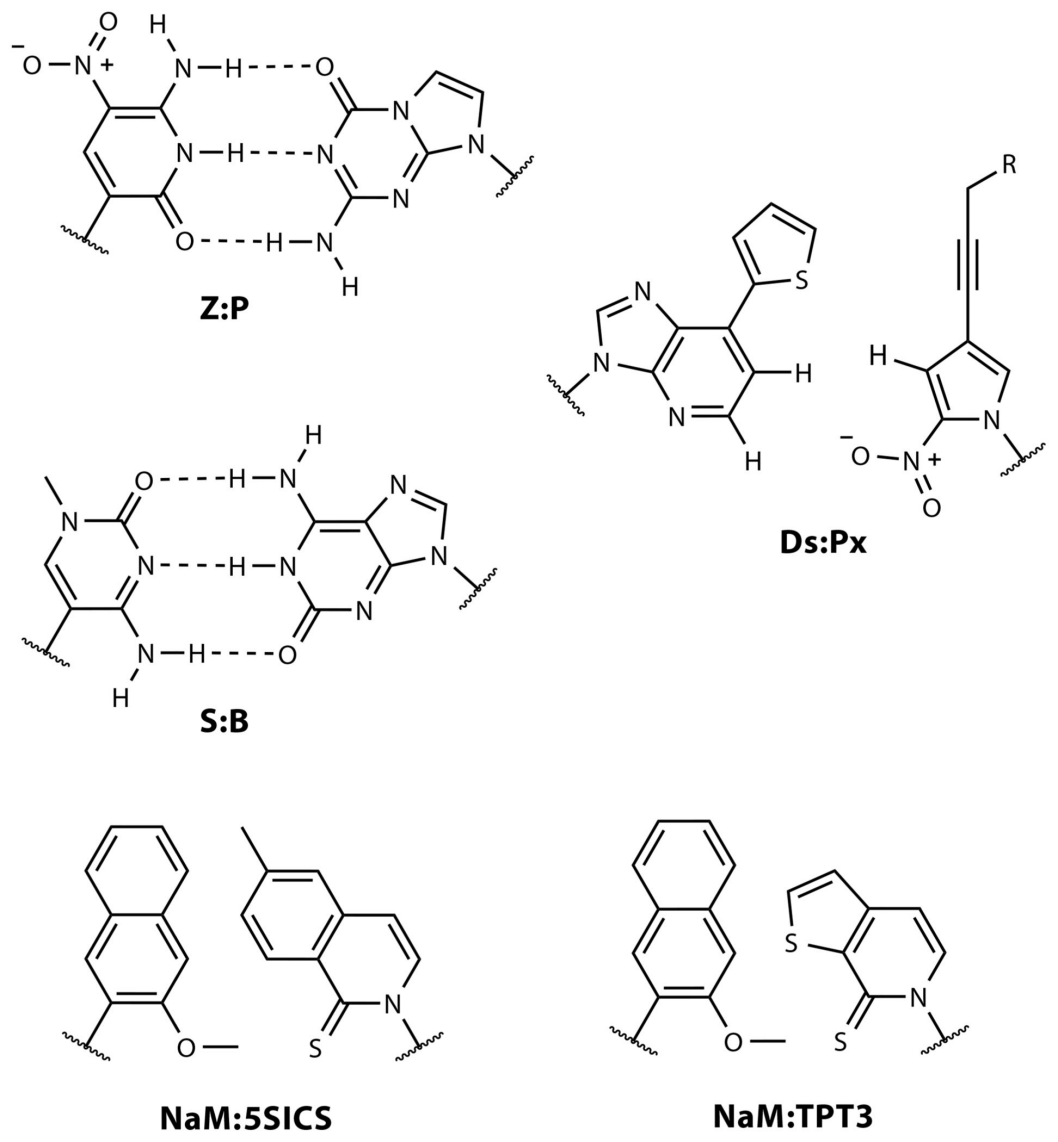
Unnatural base pair (UBP) chemistries. The UBP design concepts shown are H-bond reassignment (Z:P and S:B; *left*), shape complementarity (Ds:Px; *right*), and hydrophobic interactions (NaM:5SICS and NaM:TPT3; *bottom*).

UBP designs fall broadly into three categories, and all three have been efficiently replicated in vitro. One design, pioneered by Benner and colleagues (8, 101, 127), builds on purine- and pyrimidine-isosteric heterocycles, in which the hydrogen bond donors or acceptors have been scrambled to yield four potential new base pairs. Among these UBPs with alternative H-bond patterns, the P:Z pair is the most extensively characterized, closely followed by the B:S pair; these two pairs have been combined with the natural pairs in a new eight-letter hachimoji alphabet (42). An alternative approach from the Hirao group uses base pairs interacting mostly via shape complementarity, with the most promising results obtained with the Ds:Px pair (40, 124). The third type of UBP, developed by Romesberg and colleagues (74), relies on hydrophobic interactions and shape complementarity, with 5SICS:NaM and TPT3:NaM as the best-characterized pairs (57, 66) (Figure 3).

UBPs from all three base pairing types can be replicated with high fidelity in vitro, as demonstrated in polymerase chain reaction (PCR) amplifications of appropriate templates (51, 68, 126). However, for in vivo applications, only the hydrophobic d5SICS:dNaM and dTPT3:dNaM pairs have been explored to date. Despite their divergent structure from the natural bases, DNA templates containing hydrophobic base pair variants, such as d5SICS:dNaM, are processed by natural polymerases in vitro, with only slightly lower fidelities compared to natural substrates for the best-processed template sequences (68). Higher fidelities of up to 99.9% and reduced sequence dependency can be achieved by employing binary polymerase blends such as OneTaq to optimize the ratio of synthesis and repair via exonuclease activity in the test tube (66). Interestingly, while these hydrophobic bases intercalate within the DNA duplex (67), when replicated, the polymerase active site appears to enforce the natural edge-on structure (9), which may be a prerequisite for replication. Indeed, using a less intercalating UBP such as dNaM paired with the less aromatic dTPT3 further improves both incorporation and extension past the UBP (57). Sequence-dependent replication efficiencies are also observed for the dDs:dPx pair; however, these require at least six standard bases between subsequent Ds for successful replication (51). The reasons why consecutive hydrophobic bases are poorly replicated are not well studied but may include higher structural flexibility (due to an absence of interstrand H-bonding); general distortions of the double helix (72); and potentially a tendency to form looped-out structures by interleaving self-pairs, as has been suggested for the hydrophobic universal base 5-nitroindole (63).

Replicating hydrophobic UBPs in vivo requires an endogenous *E. coli* polymerase or introduction of specific polymerases, such as the Klenow fragment of *E. coli* DNA polymerase I, that are capable of replicating the d5SICS:dNaM pair in vitro (98). Although both d5SICS:dNaM and dTPT3:dNaM were successfully replicated in *E. coli* on a plasmid where the UBP is located in a fragment designed to be processed by DNA polymerase I (65, 131, 132), it was later shown to be DNA polymerase II and DNA polymerase III that were critical for replication in vivo (55). This clearly demonstrates the feasibility of in vivo replication of UBPs integrated into natural DNA. Major challenges to address will include the incorporation of multiple UBPs and measures to prevent UBP loss over time, which may closely correlate with the availability of unnatural triphosphates in vivo (65).

Although they have not been probed for in vivo applications to date, the Benner group’s dZ and dP nucleobases are promising candidates for the replication of multiple UBPs in series, as they are more compatible with the natural DNA architecture due to their nearly isosteric structure and similar H-bonding recognition principles (34). Indeed, they are recognized as substrates by members of both the A and B polymerase families (128). However, the polymerases differ in their efficiency and fidelity when dealing with the unnatural bases, especially when encountering multiple UBPs in series. Notably, *Taq* and Phusion polymerases are, without additional engineering, capable of replicating DNA containing stretches of up to four copies of these unnatural nucle-obases with error rates as low as 0.2% under optimized conditions (125). The major route for base pair loss is the pH-dependent mispairing of dZ with dG. Importantly, the mutation rate is bidirectional, i.e., dZ can also be gained, yielding an evolvable six-nucleotide system (125). Based on these promising results, it will be very interesting to evaluate the replication of this six-letter alphabet in vivo. The ability of polymerases to copy stretches of up to four unnatural bases should in principle allow for the replication of plasmids with a six-letter alphabet (125).

#### 3.2.1 Availability of unnatural triphosphate nucleobases in vivo

Both replication and transcription of UBPs require the presence of the corresponding nucleoside triphosphates at sufficiently high concentrations in the cell. As a reference, physiological concentrations of the natural deoxyribonucleoside triphosphates (dNTPs) range from 5 μM for dGTP to 37 μM for dTTP (113). In the absence of established metabolic pathways to generate the desired unnatural nucleotides directly in vivo, growth media need to be supplemented with presynthesized unnatural nucleosides or nucleotides, and these require efficient cellular uptake.

The most explored route in *E. coli* relies on direct administration of the triphosphates to the growth medium and uptake via specialized transporters like the nucleoside triphosphate transporter of the algae *Phaeodactylum tricornutum* (PtNTT2). This strategy enabled the import of d5SICS and dNaM triphosphates and allowed for the propagation of a single UBP over several generations in vivo, generating the first semisynthetic organism with an expanded genetic alphabet (65). Yet some challenges remain: The rapid dephosphorylation of triphosphates both in the media and in the cytosol resulted in intracellular depletion and UBP loss during replication (65), and transporter expression appeared to be toxic and thus inhibited growth. Strategies for increasing triphosphate availability include (*a*) supplementation of the medium with potassium phosphate, which acts as a competitive inhibitor for triphosphate-degrading phosphatases (65); (*b*) chemical modification of the unnatural nucleotides to increase phosphatase resistance but retain polymerase recognition (e.g., via substitution of oxygen with a difluoromethylene unit between the β- and γ-phosphate groups for dTPT3; 29); and (*c*) transporter engineering to reduce toxicity and increase expression (and thus nucleotide import) via removal of an N-terminal signal peptide and chromosomal integration (131).

A very promising approach for selection and optimization of nucleoside triphosphate transporters was developed recently for dTTP uptake (81). Transporters were chromosomally integrated in place of ompT, an outer membrane protease, and uptake of the triphosphate was coupled with cell survival in strains auxotrophic for dTTP. This design resulted in the discovery that the nucleoside triphosphate transporter from *Thalassiosira pseudonana* (TpNTT2) imports triphosphates (including some unnatural xNTPs) more efficiently and is less toxic for *E. coli* than PtNTT2.

Ideally, however, unnatural nucleobases would be administered as nucleosides, which are thought to enter cells via either passive diffusion or active transport by bacterial nucleoside transporters such as NupC or Tsx (17, 129). Once in the cytoplasm, stepwise phosphorylation by kinases of the nucleotide salvage pathway would be required to generate the nucleoside triphosphates. Rate limiting in the phosphorylation cascade is generally the first step in generating the 5′-monophosphate, and this step is catalyzed by deoxynucleoside kinases (2). While most organisms possess highly specific variants of this kinase, *Drosophila melanogaster* has a variant with broader substrate specificity, which can phosphorylate all four natural and various unnatural nucleosides, including analogs of d5SICS and dNaM (123), as well as dP and its partner dZ upon introduction of a single point mutation (15). The remaining steps to generate the di- and triphosphate, catalyzed by mono- and diphosphate kinases, respectively, have not been thoroughly characterized, but these enzymes are generally thought to be more promiscuous than deoxynucleoside kinases. Indeed, *E. coli* nucleoside diphosphate kinase accepts dZDP and dPDP as substrates to generate triphosphates that are directly utilized by DNA polymerases (73). Thus, a system may be envisioned whereby endogenous, engineered, or recombinant kinases generate the required triphosphates directly from the unnatural nucleosides. This alternative approach involves considerably more cellular engineering but also represents the first step toward engineered in vivo biosynthesis and processing of unnatural nucleobases and fully autotrophic future organisms with expanded genetic alphabets.

#### 3.2.2 Transcription of unnatural base pairs

T7 RNA polymerase (T7Rp) accepts various unnatural nucleobases as substrates, including 5SICS and NaM (99), as well as Pa and Ds (39). Similarly, templates containing up to three copies of dZ and dP (54) or an eight-letter genetic alphabet employing the Z:P and B:S pairs have been transcribed, the latter requiring an engineered T7Rp variant (42). Despite reduced in vitro transcription efficiency, recombinant expression of T7Rp in *E. coli* proved sufficient to generate mRNA from a plasmid encoding sfGFP with a single copy of the unnatural nucleobase dNaM for subsequent protein synthesis (see below), provided that the corresponding unnatural ribonucleoside triphosphates were added to the media in the presence of the engineered nucleotide transporter (132). Furthermore, *S. cerevisiae* RNA polymerase II also accepts NaM and TPT3 as substrates to produce full-length RNA transcripts, with considerably higher selectivity against natural nucleotide misincorporation if dTPT3 is present in the DNA template than if it is absent (79). This result paves the way for transcription of UBPs using endogenous RNA polymerases in both prokaryotes and eukaryotes.

#### 3.2.3 Translation of unnatural base pairs

In the early 1990s, the Benner group first demonstrated successful translation of iso-C and iso-G UBPs in vitro, observing incorporation of the uAA iodotyrosine with yields of 90%, exceeding those resulting from amber suppression (7). Thus, the ribosome is clearly capable of translating UBP-containing codons, provided that a specifically pairing tRNA is present. Building on this result, the Hirao group transcribed and translated the ds:dy UBP in a one-pot reaction in vitro to produce human Ras protein containing the uAA 3-chlorotyrosine (41), demonstrating that the ribosome is also able to process UBPs based on shape complementarity.

Recently, UBP translation of sfGFP was described in vivo in *E. coli* by Romesberg and colleagues (132) from a plasmid containing a single copy of the dTPT3:dNAM UBP, combining all previously identified requirements—heterologous expression of an optimized nucleoside triphosphate transporter, T7Rp, and addition of the unnatural (d)NaM and (d)TPT3 triphosphates to the growth media—with the addition of a plasmid coding for an engineered tRNA containing TPT3 in the anticodon loop. Moreover, introduction of an orthogonal pair of TPT3-containing tRNA and a corresponding tRNA synthetase supported incorporation of a uAA to truly expand the genetic code with the newly generated A(NaM)C or G(NaM)C codons.

Theoretically, the resulting six-letter genetic alphabet gives rise to 152 new codons. However, an initial screen of possible codons containing a maximum of one UBP identified just nine that resulted in successful protein production, suggesting constraints on UBP usage, such as a requirement for these UBPs to be located at the second codon position and the presence of at least one G:C pair (30). Yet those nine codons were decoded with high efficiency (96%) (30), demonstrating the potential of the UBP approach for genetic code expansion. These results are a promising start toward achieving the ambitious goal of creating semisynthetic organisms with divergent six- or even eight-letter genetic alphabets.

### 3.3 Synthetic Genetics: Xeno-Nucleic Acids

**Figure 4:**
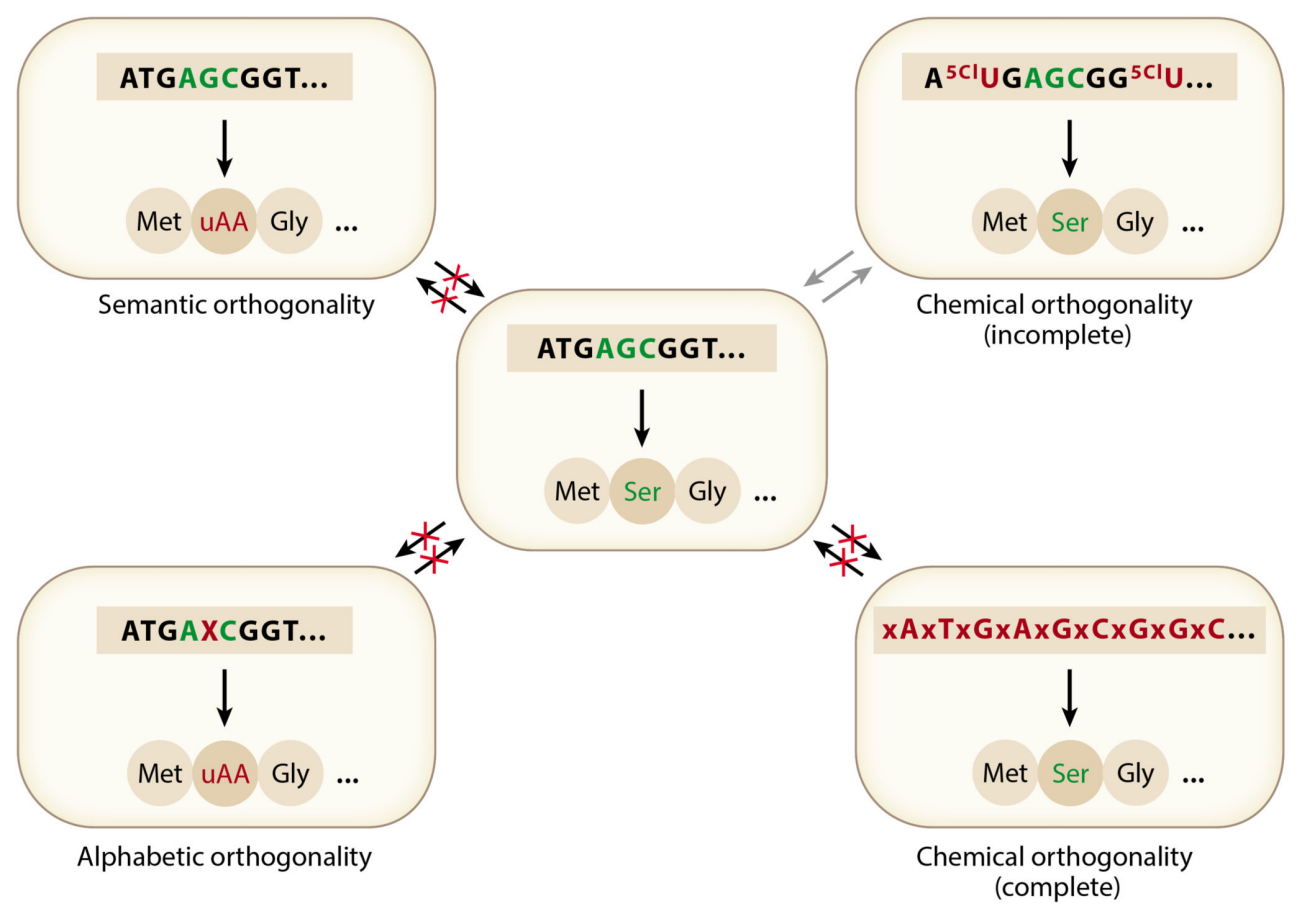
Orthogonality concepts. Genetic containment, i.e., inability to transfer genes to and from natural *E. coli* cells (*center*), may be achieved via semantic orthogonality (code reassignment; *top left*), alphabetic orthogonality (genetic alphabet expansion; *bottom left*), or (stereo)chemical orthogonality [(stereo)chemical divergence of genetic polymers; *right*].

Beyond such semantic or alphabetic orthogonality, one might ask if other forms of orthogonality might be developed, e.g., based on chemical divergence of the genetic system. One such approach has been pursued by Marlière and colleagues (71), who developed an *E. coli* strain in which dT was replaced genome-wide by the isostere 5-chlorouracil (5CU) and that, through continuous growth and evolution in the presence of 5CU, became dependent on it for growth. Due to the close similarity of T and 5CU, it is likely that this approach is only a first step to full genetic isolation (incomplete orthogonality; Figure 4), likely requiring a further expansion of the chemical divergence of such genomes.

More radical variations to the canonical chemistry of nucleic acids have been explored in efforts to define and understand the fundamental chemical and physicochemical parameters that underpin their capacity for genetic information storage and propagation (108). Apart from illuminating the profound influence of nucleic acid chemistry on function, these studies have yielded a large number of variations on the canonical (deoxy)ribonucleic acid structure used in nature that are (to varying degrees) capable of specific homo- or heteroduplex formation, genetic information storage, propagation, and evolution. These synthetic modified nucleic acids include an ever-increasing range of modified nucleic acid scaffolds, comprising sugar ring congeners and altered backbones (even including completely uncharged backbone chemistries) (32). For simplicity, we refer to all such synthetic modified nucleic acids as xeno-nucleic acids (XNAs), even if some chemical variations, such as 2′-*O*-methyl (2’OMe)-RNA or α*P*-S (phosphorothioate), can occur in nature (20, 118).

In recent decades, there has been growing interest in XNAs for applications in biotechnology, nanotechnology, and medicine, in particular as scaffolds for the emerging field of nucleic acid therapeutics. Several XNA modifications have already been approved for human use by the US Food and Drug Administration and confer beneficial physicochemical, pharmacokinetic, and pharmacodynamic properties, including biostability due to nuclease resistance, increased in vivo half-life, and increased antisense-binding affinity in the case of antisense oligonucleotides (ASOs) (114). However, the scope of XNAs is potentially much wider, as the capacity of nucleic acids (including XNAs) to fold into defined three-dimensional shapes allows the de novo discovery of ligands (XNA aptamers), catalysts (XNAzymes), and defined nanotechnology objects (105) and devices, raising the possibility that these too can be applied clinically.

#### 3.3.1 Xeno-nucleic acid ligands (aptamers)

XNA aptamers have been discovered for a range of XNA chemistries through the same process used for DNA and RNA aptamers, systematic evolution of ligands by exponential enrichment (SELEX) (122). In the case of XNAs, SELEX of XNA aptamers or XNAzymes (106) relies on the synthesis of (XNA) libraries by (engineered) polymerases (45), selection of the desired phenotype through physical separation of successful sequences, reverse transcription by (engineered) reverse transcriptases (RTs), and amplification for subsequent sequencing or entering a new selection cycle. So-called slow off-rate modified aptamers (SOMAmers) bearing (hydrophobic) substituents at the pyrimidine C5 position enabled discovery of aptamer arrays against a wide range of targets that are promising for diagnostic biomarker monitoring (36). However, for fully backbone-modified XNA aptamers with sugar congeners like α-L-threofuranosyl nucleic acid (TNA), 2′-fluoro-arabinonucleic acid (FANA), and 1′ 5′-anhydrohexitol nucleic acid (HNA), engineered polymerases are required to polymerize some xNTPs, i.e., tNTPs (78), faNTPs (82), and hNTPs (82), respectively. The full SELEX cycle also requires RTs to convert XNA back into DNA. While some XNA chemistries, like TNA (23) and FANA (119), can be reverse transcribed by naturally occurring RTs or their engineered forms, such as Moloney murine leukemia virus (M-MuLV) RT (available commercially as Superscript I-IV) or Bst DNA polymerase (available commercially as RTx) (80), most XNA chemistries require bespoke RT engineering to enable efficient reverse transcription (46, 82).

TNA aptamers were selected to bind programmed death-ligand 1 (58) or HIV-1 RT (24, 75), as well as small-molecule targets like ochratoxin A (84) and adenosine triphosphate (130). FANA aptamers binding HIV-1 RT (3), integrase (91), and the receptor-binding domain of the SARS-CoV-2 Spike protein (4) have been developed, showcasing once more the ease of aptamer selection against naturally nucleic acid–binding proteins. In addition to the original anti-HIV TAR and anti-Hen Egg Lysozyme aptamers (82), HNA aptamers with a high affinity for vascular endothelial growth factor (VEGF) have recently been described (27). Finally, even uncharged backbone chemistries like *P*-alkyl phosphonate nucleic acids (phNAs) have been evolved into aptamers (5).

#### 3.3.2 In vitro applications of xeno-nucleic acids: XNA catalysts (XNAzymes)

Nucleic acids can fold into three-dimensional structures in a way that renders them active catalysts. A classic example is the 10–23 DNAzyme, which cleaves RNA in a Mg^2+^ -dependent manner (94). Because of its potential promise as a gene-silencing agent, the 10-23 DNAzyme has been intensively studied. However, it has broadly failed to have clinical impact, possibly due to its dependence on unphysiological concentrations of Mg^2+^ for both folding and catalysis. Indeed, a recent nuclear magnetic resonance analysis of the 10–23 structure confirmed its structural and conformational plasticity and the central role of Mg^2+^ ions in shaping the transition from the inactive precat-alytic to the catalytic complex (12). Chemical modifications have been explored in an effort to enhance the properties of the 10–23 DNAzyme. For example, the study discussed above identified a 6-thio-dG14 modification that increased the cleavage rate (*k*_obs_) approximately sixfold. Similarly, FANA modifications at G2 and U8 in the catalytic core and full FANA-substitution of the RNA-target binding arms, in combination with two additional flanking TNA nucleotides, yielded a modified X10–23 with improved catalytic rate, biostability, and in vivo gene-silencing activity (120). Fully modified XNAzymes have also been obtained, including functionally analogous RNA-endonuclease FANAzymes (109, 119). In both cases, antisense effects likely make significant contributions to the apparent in vivo gene-silencing activity of the chimeric DNA-FANA (X10-23) or of all-FANAzymes (107, 110), as both DNA and FANA (and in particular mixed FANA-DNA oligonucleotides; 103) are able to efficiently recruit RNase H (the cellular enzyme that cleaves DNA-RNA heteroduplexes) (18). These effects may be disentangled by the study of RNA endonuclease XNAzymes elaborated in chemistries that do not recruit RNase H, such as 2’OMe-RNA (2′OMezymes) (33) or TNAzymes (121). With increasing structural and functional understanding of RNA endonuclease XNAzymes, there is hope that their strengths, i.e., their easy programmability, single-nucleotide specificity, and small size (enabling chemical synthesis) can be leveraged for their development as effective gene-silencing agents.

#### 3.3.3 In vivo applications of xeno-nucleic acids

ASOs are emerging therapeutic modalities that have also greatly benefited from expanded nucleic acid chemistries. Natural chemistry-based RNA ASOs like small interfering RNAs (siRNAs), siRNAs with several chemical modifications that confer nuclease resistance, and DNA-based—usually phosphorothioated (α*P*-S)—ASOs, sometimes with flanking chemical modifications to make biostable gapmers, are part of the established ASO technology toolbox (21, 114). DNA gapmers recruit RNase H to degrade bound RNAs and rely on a central (α*P*-S)-DNA stretch crucial for RNase H recognition. The only XNA chemistry found to be able to elicit RNase H cleavage on a bound RNA is FANA (see above), but it is not as nuclease resistant as α*P*-S-DNA (18). Many other, more divergent chemistries, such as 2′-fluoro, 2′OMe, 2′-*O*-(2-methoxyethyl) (MOE), locked or bridged nucleic acids (LNAs/BNAs), and constrained ethyl nucleic acid (cEt), including fully modified (α*P*-S)-MOE-RNA and phospho-rodiamidate morpholino oligomer (PMO) ASOs, are used in the development of novel antisense medicines (114).

The repeating negative charges on the nucleic acid backbone inhibit passive transport across cell membranes. Recent endeavors to address this challenge target the phosphodiester backbone directly and introduce uncharged linkages, as in alkyl phosphonate nucleic acids (phNA) and phosphoryl guanidine oligonucleotides (PGOs) (49) or even positively charged guanidine-bridged nucleic acids (GuNA) (102, 104). While these approaches are elegant solutions to a physicochemical problem, they are still limited by larger problems like antisense-binding affinity to RNA targets, cost-efficient availability of starting materials, and ease of synthesis. While many of these chemistries have been explored by solid-phase synthesis, increasing success in polymerase engineering has enabled enzymatic synthesis of several of these chemistries, such as 2’OMe-RNA, phNA, MOE-RNA, and LNA (or blends there of) (5, 33, 38, 44), potentially enabling future rapid iterations of ASO sequence and chemistries to accelerate discovery.

#### 3.3.4. Toward chemical orthogonality

XNAs introduced into cells (e.g., as described above for UBPs) could increase cellular function and information content in several ways. Alternative backbone chemistries, when synthesized inside the cell, could be designed to form specific XNA aptamers, XNAzymes, or ASOs (see above) that affect cellular regulation, e.g., by modifying protein interactions or modulating gene expression. XNA backbones (such as α*P*-S) can also affect decoding efficiency, especially on the level of translation (50, 52). However, a fundamental issue with most XNA chemistries is that, while they are divergent from the canonical DNA and RNA chemistry, they may be insufficiently orthogonal to avoid interference with the cellular nucleotide metabolism. Indeed, while UBPs appear to be largely nontoxic, the case of genomic rNTP incorporation indicates that even sporadic incorporation of noncognate nucleotides can lead to genetic instabilities (133). One potential approach to isolate XNAs further from the cellular metabolism would be to increase chemical divergence, and thereby orthogonality, by engineering XNA poly-merases for the acceptance of alternative leaving group chemistries (37) or uncharged backbone chemistries (5). Orthogonal in vivo XNAs may also require orthogonal replication, e.g., via the recruitment of a dedicated polymerase to the specific locus.

An interesting strategy for bio-orthogonal genetic isolation exploits stereochemical rather than chemical divergence. Orthogonal genetic mirror-image elements would be exceptionally suited for biocontainment strategies, since there is little crosstalk between proteins or nucleic acids of opposite chirality (6, 76). Progress in this area is based on the development of tools for the templated synthesis, replication, and evolution of L-DNA and -RNA (the mirror-image form of the naturally occurring D-DNA and -RNA) by mirror-image polymerases (elaborated in D-amino acids) (28, 97). A clear advantage of this method is that—provided the mirror-image enzymes can be made synthetically (or by a future mirror-image ribosome)—no further engineering is required. However, at the cellular level, it is unclear how such enantiomeric orthogonality could be established in stages, which would require either the full implementation of a mirror-image cellular metabolism (or at least genetic system) or the engineering of dedicated cellular components, e.g., polymerases, for the polymerization and processing of L-DNA and -RNA.

**Figure 5:**
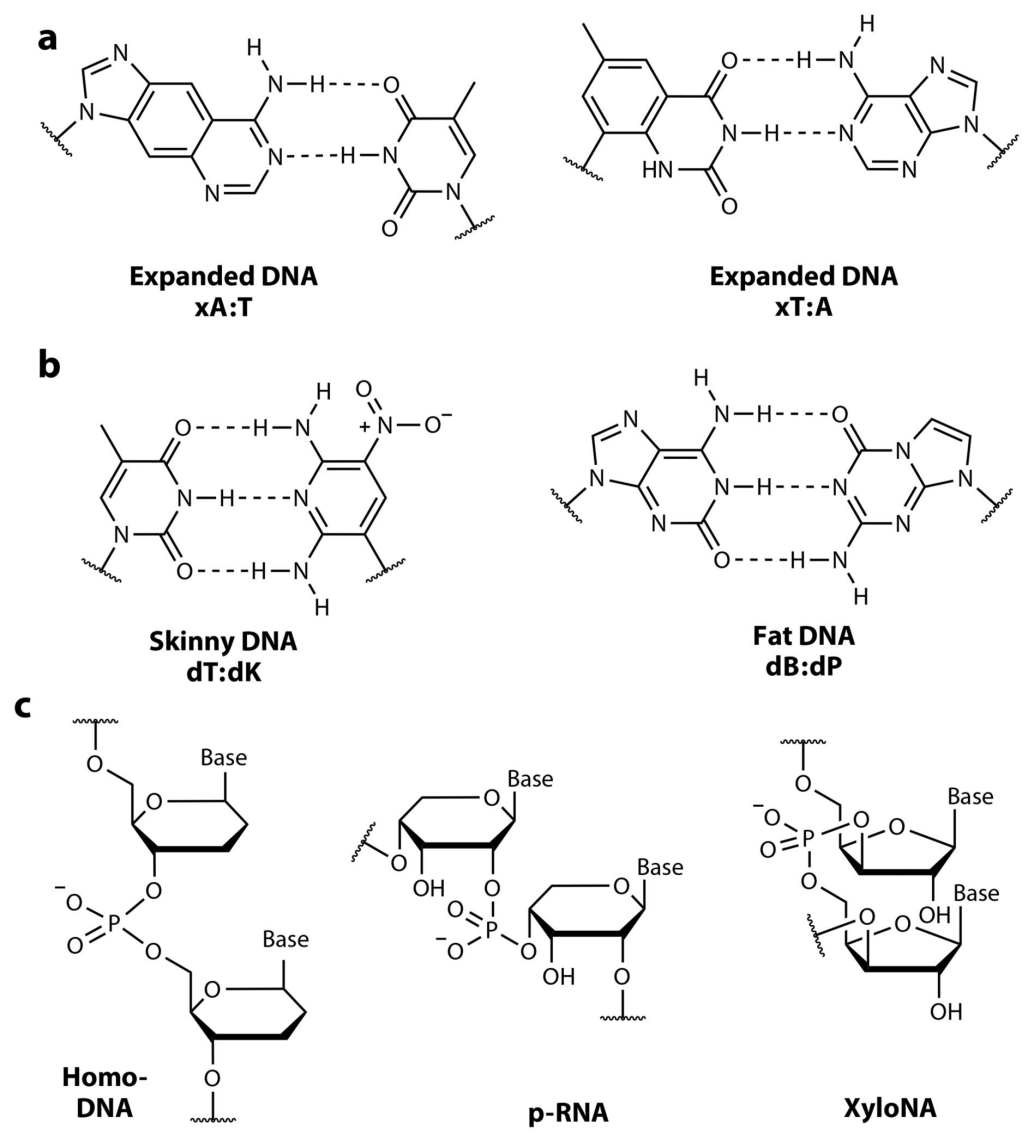
Xeno-nucleic acids (XNAs) with potential for chemical orthogonality. (*a*,*b*) Two different approaches to steric orthogonality either by (*a*) expansion of natural bases or (*b*) new base pairs between either pyrimidine-like (skinny) or purine-like (fat) DNA bases. (*c*) Chemical orthogonality.

Other approaches to further chemical orthogonality, for example, based on XNA chemistries like homo-DNA, pRNA, or XyloNA (14, 26, 53) (Figure 5), which are unable to cross-pair stably with the natural nucleic acids, seem possible but would be even more challenging to implement. Steric orthogonality, whereby chemical orthogonality arises from significant steric divergence from the natural nucleic acids, such as in the expanded nucleic acids (xDNA, yDNA) of Kool and colleagues (60, 61, 64) or the fat and skinny DNAs of Benner and colleagues (43), may be another possibility, but these chemistries would likely not be fully orthogonal, as both x(y)DNA and fat (skinny) DNA maintain pairing with the natural bases. In all of these cases, efficient enzymatic synthesis and replication, let alone translation, remain to be established.

Thus, in terms of chemical orthogonality, the challenge is paradoxical in the sense that chemistries that can be accepted and processed by the extant cellular machinery (to some extent) are likely toxic, while those that diverge sufficiently to be fully orthogonal require such extensive cellular re-engineering as to be currently impractical. It would therefore seem that chemical, steric, or stereochemical orthogonality, i.e., orthogonal genetic systems based on XNA or mirror chemistries, may find more immediate utility in in vitro applications and in the context of the emerging field of synthetic biomaterials and cells (1, 90). Synthetic cells are based on a much-simplified synthetic cellular machinery, which would be much more amenable to wholesale engineering or replacement. Thus, synthetic cells comprising chemically orthogonal XNA genomes and/or transcripts might provide both a useful test bed for the various synthetic genetic concepts outlined above (including the step-wise development toward in vivo applications), as well as providing an opportunity to implement additional layers of control and added functionalities in synthetic cells and biomaterials.

## 4 Conclusion

By expanding the central dogma and extending the boundaries of life both at the informational and at the chemical level, the concepts outlined in this review promise not only to augment our abilities for biological engineering, but also to extend them to studying and understanding biological function and evolution at a deeper level by opening up fundamental aspects of the informational organization and chemical makeup of life for investigation.
